# Psychological Impact on Parents of an Inconclusive Diagnosis Following Newborn Bloodspot Screening for Cystic Fibrosis: A Qualitative Study

**DOI:** 10.3390/ijns5020023

**Published:** 2019-06-11

**Authors:** Faye Johnson, Kevin W. Southern, Fiona Ulph

**Affiliations:** 1 Division of Psychology & Mental Health, School of Health Sciences, Faculty of Biology, Medicine and Health, University of Manchester, Manchester Academic Health Science Centre, Oxford Road, Manchester M13 9PL, UK; 2 Institute of Translational Medicine, University of Liverpool, Liverpool L12 2AP, UK

**Keywords:** newborn screening, qualitative, communication, CFSPID, uncertainty, impact, psychological, parent

## Abstract

Genetic results of uncertain clinical significance are being returned to parents following newborn screening, representing a paradigm change in how society considers health and illness. ‘Cystic Fibrosis screen positive, inconclusive diagnosis’ (CFSPID) is a designation given to newborns with a positive screening result for, but not a definitive diagnosis of, cystic fibrosis. We explored the psychological impact of receiving a CFSPID result on parents. Five semi-structured interviews were conducted with eight parents whose children have CFSPID. Interpretative phenomenological analysis identified these themes: “The way we were told”: ‘diagnosis as a traumatic event’ focused on how parents were distressed and dissatisfied by the initial screening result communication, ‘Facing and challenging traditional ideas about health and illness’ explored the emerging problem of how CFSPID does not fit the commonly accepted medical model, and ‘Making certainty out of uncertainty’ explored the varying strategies parents developed to adapt to the uncertainty regarding their child’s prognosis. Findings suggest that CFSPID results caused parents’ distress, initiated with the first communication of the result and persisting thereafter. Our data suggests approaches to the delivery of CFSPID results that may reduce the impact. Work is needed to close the gap between healthcare advances and societies commonly held medical model.

## 1. Introduction

Newborn bloodspot screening (NBS) allows for early identification and management of genetic conditions [1]. NBS can now identify abnormalities of uncertain clinical significance [2]. ‘Cystic fibrosis screen positive, inconclusive diagnosis’ (CFSPID) is a designation given to infants with a positive NBS result, but not a definitive diagnosis of cystic fibrosis (CF). Evidence suggests the number who go on to develop some form of CF varies based on the NBS protocol, but at present, no clear indication can be given to parents about the likelihood that their child will develop CF, the type of CF, or when they might show symptoms [3]. Both the ECFS Neonatal Screening Working Group and the CFF have produced guidance on clinical management which stress the importance of clear communication [4,5]. However, the guidance does not go any further or provide clear guidance on what the content of that communication should be or how to ensure it is clear. This is important as research with parents who received NBS carrier or false positive results suggests medically ‘benign’ results may have a significant psychological impact [6,7], which is often moderated by communication efficacy.

Furthermore, although it is acknowledged in the field that CFSPID designation may cause uncertainty, a state which is known to cause distress, relatively little research has explored the psychological impact of CFSPID for parents [8,9]. A questionnaire study suggested that parental distress over CFSPID may be of a similar level to parents of children with definitive CF diagnoses [10]. However, questionnaires may not capture psychological complexity due to closed questions. Massie and Gillam [11] considered various management options in CFSPID. Potential harms include over-medicalisation and altered parental attachment. Nevertheless, their arguments are hypothetical. One qualitative study has confirmed these potential harms are experienced by parents in a setting where screening is mandatory and there may be health insurance implications [12], The purpose of this paper was to conduct further, in-depth research to underpin communication guidance. This paper aimed to address the gap in communication guidance via an in-depth exploration of the psychological impact of a CFSPID designation on parents.

## 2. Materials and Methods

### 2.1. Study Design

A qualitative study using interpretative phenomenological analysis (IPA), which is an in-depth method which is well suited to health psychology [13] and suitable for novel research topics and small samples [14]. IPA was chosen as it enables the in-depth study of lived experience of small homogenous groups [14] and acknowledges that the analysis involves the researchers’ interpretation of the participants’ interpretations of their experiences. This is useful when the participants themselves may not use psychological terminology or theories to interpret their experience, but one wants to create an overall understanding of psychological factors in an experience. Inclusion criteria were that parents had received a CFSPID result following NBS via the clinic. Parents who did not speak fluent English were excluded due to funding constraints. Parents were excluded if their child had died. Ethical approval was obtained from the NHS Health Research Authority (North West—Preston Research Ethics Committee on 20 February 2018, No. 219764).

### 2.2. Recruitment and Consent

Convenience sampling was used whereby parents who had received a CFSPID designation for their child were recruited via a CF clinic in North West England. IPA is typically conducted with small sample sizes, congruent with its emphasis on individual meaning [15]. The decision to cease recruiting was based on IPA guidelines to provide sufficient time within the project to enable in-depth analysis of the data and suitable sample sizes for projects, an ongoing review of the issues covered in interviews to establish data sufficiency, and the rate of return of forms from potential participants.

Study packs were distributed to parents by clinic staff. Parents returned a “consent to contact” form if they were interested in hearing more about participation. They were then called by F.J. to discuss the interview and set an interview time if wanted. Before the interview started, salient aspects of the participant information sheet were discussed, and participants could ask questions. If they agreed to proceed, they were asked to sign a consent form.

### 2.3. Participants

Three couples and two mothers participated. The parents could choose whether to be interviewed together and all couples requested this. All participants were interviewed once. Parent characteristics can be seen in Table 1. Children were age 2–8 years, with no siblings with CFSPID.

### 2.4. Interviews

The semi-structured interview schedule was developed with CF and qualitative NBS research experts, guided by literature (available online). Topics included: initial result communication, impact of CFSPID on identity and parental role, telling others/the child about CFSPID, role of healthcare professionals (HCPs), impact of CFSPID knowledge, and suggestions. Questions were open, with flexible prompts to elicit more detail. Drawing tasks were used to help parents consider abstract concepts. These included a series of concentric circles to enable parents to convey how close various people who were involved in this time were to them and reflect on conversations. It also involved stick diagrams of people depicting continuums from healthy and unhealthy to enable them to reflect at different time points how they viewed their child (see interview schedule). The verbal responses were analysed, rather than the drawings. Parents were interviewed at home by F.J., with interviews lasting 50–150 min. Audio recordings of interviews were transcribed verbatim, removing identifiers. Pseudonyms were used.

### 2.5. Ethical Approval

Data were analysed using Interpretative Phenomenological Analysis (IPA) [16]. Each transcript was analysed separately before identifying patterns across the dataset. Analysis was supervised by an experienced IPA researcher. Coding was checked for completeness and representation of the dataset as a whole. Data exemplars were selected from each participant to illustrate how each concept applies to each participant (case within theme), in line with IPA guidance for a sample this size [16]. Due to the researcher’s own background and the focus of much of their training in psychology and health services, it is possible that CFSPID was approached as a problem to be solved, i.e., expecting that parents would have issues with their child’s CFSPID designation, where perhaps they may not have done. These preconceptions may have influenced participants’ responses, for instance through small cues during the interview process. The researcher’s interpretative analysis of the interviews may to some degree have been conducted through this ‘problem solving’ lens.

## 3. Results

### 3.1. “The Way We were Told”: Diagnosis as a Traumatic Event

#### 3.1.1. Intrusion and Taking Away of Control

All parents described similar experiences of unexpected visitors to their homes, interrupting everyday life with a newborn. Sue, whose husband was working away, described this:

*SUE: “A lady come to the door, and she just said […] she’d come in to talk to us, I’d no idea who she was or anything like that, so she just came in, and then she sat me down.”*

The behaviour of the professional felt intrusive and transgressive—unlike usual interactions, the visitor came in without asking and without Sue knowing her identity. Sue appears not to have had control in this moment, which immediately raised alarm—Sue’s husband *“had to rush home.”*

Guy, whose wife Liz was out at the time, described the visitors as *“[…] insistent, y’know, get your wife back home”*. Guy instantly felt something was wrong:

*GUY: “Got a knock on the door, two health visitors come round, unannounced, and I know from experience when people come around unannounced it’s not great news.”*

Molly, who was at home with her two young daughters, described her experience:

*MOLLY: “The door went, in walked two health visitors to basically tell, tell us the news […] and especially because I was like breastfeeding as they arrived as well, you just, you feel very vulnerable […] it’s just not a nice thing to have strangers turn up at your house.”*

Again, they came uninvited, immediately creating a power imbalance. Molly explained that she felt vulnerable—arguably the antithesis of what healthcare should feel like.

Sadie also experienced the timing of the visit to be inappropriate and intrusive:

*SADIE: “kids’ dad was saying ‘Well can’t you come back?’ and she was going ‘No, I need to come in now’… cos I was having a sleep with Mia, she went ‘You need to wake mum up’, so we got told to come down the stairs.”*

Although medically necessary, the visitor’s insistence on coming in takes control away from Sadie and her partner and connotes a sense of worrying urgency.

For another parent, the sense of alarm, vulnerability, and loss of control was harmful:

*ANNA: “They said ‘Can you talk? We need to speak to you. Can somebody have the baby?’ […] and I said, ‘Yeah, why?’ and she said ‘Just, you need to get, give your baby give the baby to your mum.’ […] and she just sat us down didn’t she and said ‘I’ve got some [pause] bad news’, […] I honestly thought she was coming to have me sectioned.”*

The urgency of the visit, intensified by having to hand the baby over, compounded Anna’s feelings of vulnerability. Having experienced postnatal depression, Anna was frightened that she was being sectioned—an ultimate example of having control taken away. The visits, and the manner in which they were conducted—the sense of urgency, without prior warning—sent the message to parents that something was terribly wrong and impacted on their interpretations of the screening result as a serious, life-threatening diagnosis. In general, parents had strong, lasting memories of the visits as negatively affecting their experience of home life with their new baby.

#### 3.1.2. Fear, Grief, and Threat to Child’s Life

Parents varied in their willingness to discuss their psychological responses to diagnosis during the interview, some appeared more pragmatic, suggesting “*the way you find out” (Molly)* should be changed. Communication of the screening result led to all parents’ initial belief that their child had an unequivocal CF diagnosis. Liz described the fear this caused:

*LIZ: “I think it’s scary wasn’t it? Cos all of a sudden, because the way it was put across to us by the health visitors is that Ava had CF and that was final […] wasn’t a chance that she didn’t have CF so it was us trying to accept that […] it was scary, upsetting, worrying—of how we were gonna manage—A child with CF.”*

Liz, a healthcare professional with knowledge of CF, was forced to face a challenging new future. All parents knew of CF as serious, with a poor prognosis, as discussed by Sadie:

*SADIE: “The time’s gonna come when Mia’s in hospital, she’s gonna miss out on school trips gonna miss out on birthdays […] might not even have a Christmas, we don’t know.”*

Though she knows that Mia (now 8) is well, Sadie maintains that she *“is not a normal child”*, and appears to have retained the fear of her daughter’s life being severely limited: *“Is the day and the time gonna come where, y’know, it’s gonna kill us?*”

Harry interpreted his son Ben’s diagnosis through his own personal history of loss:

*“Bombshell wasn’t it? It was confusing, cos my dad died when I was 10, and I imagined Noah at that age at his brother’s funeral, and that was all that was going through my head at the time.”*

Harry emotively described his all-consuming grief, the term ‘bombshell’ connoting total devastation, illustrating how diagnosis is not received in isolation, but interpreted in the context of personal factors. The ultimate fear of a child dying was also experienced by Sue:

*SUE: “She said it was cystic fibrosis […] I just kept saying to her ‘Oh my god is she gonna die, is she gonna die?’, and she just said ‘Well I don’t, I can’t say anything like that obviously’ so she just said ‘You need to speak to someone at the hospital’.”*

The visitor’s communication took Sue immediately to fearing and begging for her child’s life. The visitor appeared to give a standardised pat response, leaving Sue’s desperate need unmet.

#### 3.1.3. Information Sufficiency

Parents unanimously felt initial information was inadequate and inappropriate. Disappointment and exasperation were palpable in their recounts:

*JIM: “We didn’t get any kind of level of information really, apart from they said that this had come back on the screening and they left a leaflet which was about CF […] that said your child might not make it to 30. And had no information other than that.”*

They were visibly frustrated about the *“very outdated”* leaflet (Molly), blaming it for the initial belief that Ruby had *“classic CF […] shortened life expectancy and all of that” (Jim)*. This is echoed by Sue, whose visitor “*was in and out within ten minutes”,* as if on an errand:

*“…and she’d gone [sigh]. So she left us with a pamphlet, it was quite old it was an old pamphlet, it does it needs a lot of updating, and when you go onto the internet and you read it up about it it’s very scary.”*

Sue was triggered to fear for her daughter’s life, yet she did not feel she was given adequate information. The phrases ‘she’d gone’ and ‘she left us’ suggest Sue felt abandoned. Although *“you try not to Google” (Liz)*, parents needed information as soon as the alarm was raised. Unfortunately, this led to inappropriate information, perpetuating diagnosis fear:

*HARRY: “I just couldn’t get my head round what it was, and I remember she said ‘Don’t go on the internet to look at it’, what a ridiculous thing to say, who’s not gonna do that? So we went on the internet obviously, and er after she’d gone, and that’s when you see things like iron lungs, average life expectancy 40, and you think [expletive], pardon my French but… I dunno, it’s just, worst night ever wasn’t it.”*

Harry suggests that parents’ strong need for information renders the suggestion of not going on the internet unrealistic. Doing this after the visitor left sounds surreptitious, suggesting a breakdown of trust between parents and healthcare professionals. Like Sue, the information exacerbated distress, leaving a vivid impression five years later. Of note, many parents reported they were signposted to the CF trust website by the hospital team and found this site was very useful, both in terms of information and peer support from other families.

Sadie’s account differed from the other participants’, as at the time of the diagnosis she was in an abusive relationship, and did not feel able to engage with the information being given:

*“It wasn’t real […] I was just numb and not understanding […] I weren’t really that much aware of what was really going on, all I was really trying to do was keep my head above water.”*

Sadie’s account highlights the issue that, in difficult circumstances, there may be barriers to understanding. Families may need additional support to understand the screening result.

#### 3.1.4. Extended Issues of Heightened Health Risk Perception

In the period following diagnosis, parents felt a range of negative emotions, such as Sue, who questioned her parental role:

*“What am I gonna do? Is she gonna be in and out of hospital all the time, is she gonna be really ill? How am I gonna cope?” […] It wasn’t so much emotional as trying to fit things in, and I think that’s the way I went for a very long time.”*

Sue was reacting to demands of managing a serious illness, causing anxiety and self-doubt, years after she was told that Tilly had CFSPID (not CF). This eclipsed her ability to process her emotional reaction. Molly and Jim had a visceral initial reaction to heightened health risk *“…cleaning constantly, I had the most horrendous eczema on my hands, because I just wanted everywhere to be clean*” *(Molly)* and were perhaps the highest seekers of ongoing reassurance from professionals that they were doing *“…the right thing” (Jim)* for 4-year-old Ruby. The assumed burden of serious illness also affected other parents:

*LIZ: “I felt really guilty for having her […] It’s just a responsibility that you’ve had this child who’s now got this horrible condition, and that we’ve made her.”*

This burden of responsibility and self-blame is still remembered vividly two years later. This extended ramification of genetic diagnosis was also felt by Anna and Harry:

*ANNA: “I used to say were me and Harry not meant to have children, cos our faulty genes clashed together […] y’know, if you’d had children with someone else you wouldn’t have had a child with CF probably.”*

The guilt and blame associated with the diagnosis caused Anna to doubt her relationship with her husband. Again, 5-year-old Ben is referred to as having CF. Anna and Harry had wanted to have another baby but, due to the diagnosis, did not go on to do so. Both have experienced mental ill health, which they attributed to the impact of the diagnosis:

*HARRY: “Everything changed for us didn’t it, everything blew up […] Looking back we probably both had depression didn’t we, at some stage over the last five years.”*

Again, Harry’s language connotes the destructive effect the diagnosis had on him and his family.

In Sadie’s relationship with her ex-husband, the diagnosis added a further stressor:

*“I honestly believe that I stayed with him because of Mia’s condition […] it was ‘can I manage raising a child with a chronic illness on my own?’”*

Like Sue, Sadie doubted her ability to care for her child. In an abusive relationship, this was exacerbated and the idea of Mia’s *“chronic illness”* further controlled Sadie into staying, facing and challenging traditional ideas about health and illness.

#### 3.1.5. Compressed Diagnostic Odyssey

Accounts of the diagnostic process highlight that it can feel overwhelming. From the initial home visit, parents are fast-tracked to a hospital appointment the next day. This sudden shift is apparent in Sadie’s account:

*“[The visitor said] ‘you’ve got an appointment 9 o’clock with the [hospital] team which is centre of excellence, they’ll be all waiting for you…’, sorry it’s getting a bit emotional […] the team were all there explaining and again I was in denial.”*

Sadie depicts a whole new cast of characters suddenly all there waiting for her, perhaps before she was psychologically ready to engage with the process and their explanations. 

Liz’s ‘compressed diagnostic odyssey’ was evident in her vivid recollection of the specific days of the week when the medical procedures took place. Her memory of *“all the different stages”* suggested an indelible memory of undergoing the process, yet she struggled to recall the specific medical terminology *“…then we went to [hospital] so they could do a more—oh what’s the word?—a different type of sweat test”.* These contrasts connote the incongruity of the medical world and her world. Sue describes her experience similarly:

*“The next day we went to the hospital and met the respiratory team, and—It placated us a lot because they told us that Tilly was CFSPID […] what they wanted us to do was come in for lots of tests and take her for a sweat test, she wanted to start her on a course of erm antibiotics straight away, and all the other erm… she needed fetal [sic] elastase tests [...] hospital over the next six weeks quite a lot, er found out that she was pancreatic sufficient so she didn’t need any enzymes, er then we put her in for a sweat test, but it was inconclusive so she’s had to have another four or five.”*

Sue was instantly immersed into a tangibly exhausting series of procedures, the unfamiliarity of the medical world connoted by her hesitative use of terminology. Sue’s ‘placation’ on learning of CFSPID reinforces the state of high-distress she had been in, like Harry, for whom the outcome was that *“I didn’t think he’d be dead by age 5 anymore”.* Indeed, although all parents described the diagnostic process as exhausting, they unanimously saw *“the hospital day” (Harry)* as a turning point, when they received adequate information and support, *“we met the consultant who was then fantastic […], and who explained the whole thing” (Jim)*.

#### 3.1.6. A Situation that is Incongruent with the Traditional Medical Model

Rather than noticing symptoms and obtaining a diagnosis, parents were suddenly given information that clashed with their experience of their child “*as far as I knew she was fit and healthy” (Sue)*, *“she’s not in hospital… she hasn’t got an open wound” (Sadie).* Guy described this:

*“It was so out of the blue, it wasn’t like she was an ill child and then somebody comes to the door and goes, she’s tested positive for this, and you’d know straight away wouldn’t you, […] that fix the jigsaw that’s, that’s what we’re dealing with here, it wasn’t like that, she was healthy—still is.”*

Guy’s jigsaw metaphor suggests his experience did not align with his expectations of the diagnostic process and was discombobulating. Anna explored the emotional impact of this:

*“They say ‘Yeah, you’ve got a lovely healthy beautiful baby boy’, and they place him in your arms and you think phew, […] everything’s alright. And then 3 weeks later you’re told it’s actually not, and that’s really hard.”*

Anna felt secure in the belief that she had a healthy child, only for this to be reversed. Anna experienced the same emotional impact as if Ben had a conclusive disease.

#### 3.1.7. Utility of Labelling

Parents gave a range of interpretations of the power of the CFSPID label. For Sue, ordinary occurrences were interpreted in the context of disease:

*“She has to be careful about where she plays, splashing in muddy puddles and things like that when she goes to play areas, if there’s someone coughing near her I have to make sure that they’re not coughing on her, cos she can pick up a cough quite easily. She seems to pick up everything that everybody else has got but, you never know how much of that is just […] child, and how much of it is what, what she’s got.”*

Sue’s account suggests a struggle to reconcile a view of Tilly as a typical child with knowledge of CFSPID. Sue appears to need to hyper-control the environment, suggesting that disease is a constant threat. Similarly, Molly remained anxious that 4-year-old Ruby’s activities were potentially *“dangerous for your lungs” (Molly)*. Inherent to these concerns is the continued threat of respiratory illness, conferred by their perception of the CFSPID label.

Harry discussed how the term ‘CFSPID’ was only introduced to him and Anna recently:

*“I never refer to him as SPID, I just say he’s got CF […] I throw in sometimes that it’s associated with a milder form, but when you’re talking to people, on the whole, they don’t know anyway, so […]”*

When asked about the effect of the new label, neither parent felt it had changed their thinking. Harry suggests that one use of a diagnostic label is communicating with others about his child. He does this by reverting to the known diagnosis of ‘a mild form of CF’.

Liz and Guy also discussed the utility of a diagnostic label as a communicative tool, such as for school, *“I don’t put it on forms or anything cos she hasn’t got a condition” (Liz).* Unlike other diagnoses, CFSPID does not allow intervention and treatment, *“You’d only put something in if there’s a practical […] but they couldn’t do anything” (Guy)*—so they have no place for it in their lives: *“The thought of it being used as a label drives me insane, I couldn’t imagine it” (Guy).* Jim also considered this issue, with a different outcome:

*[…] whether there was kind of even a consideration as to whether people need to know if their child’s got CFSPID? Erm, but I would say for us, I would still want to know—cos even though it’s not impacting on our life we’re still doing things as preventative, to make sure that she’s gonna be as healthy as possible, even though she’s not symptomatic. So there would never be an occasion where I think, ‘oh I wish I didn’t know’.”*

Jim’s hesitance suggests he may have felt uncomfortable raising this, as medicine is traditionally revered. Jim concluded that knowing about CFSPID is right for his family but acknowledged that others may feel differently. Interestingly, a healthy child in context of CFSPID becomes *“not symptomatic”,* and routine healthy choices become *“preventative”*.

Like Sue and Jim, Sadie appeared to value the greater control over her child’s health risk:

*“I feel she’s a bit more fortunate […] gets a first-class MOT every year […] anything that’s gonna go wrong […] we’ll be ahead of the game every single time.”*

Although presented as a positive aspect of CFSPID, Sadie’s consideration of what may *“go wrong”* suggests constant apprehension of illness developing. Her insights hint at a new medical model, or set of beliefs about how illness is identified and managed, based on monitoring for known risks:

*“Is it gonna cause problems is there gonna be issues, yeah, it’s like having fair skin, if you go in the sun too long you’re gonna get burnt, but if you put a factor on, y’know, you’re gonna prevent it.”*

Sadie’s analogy raises questions about predictive medicine. A genetic propensity to sunburn is not a diagnosis, whereas CFSPID carries additional meaning and effects.

### 3.2. Making Certainty Out of Uncertainty

#### 3.2.1. Liminality and Uncertainty

All parents were asked to place their child on a continuum of ‘completely healthy’ to ‘serious health condition’ and explain why. Some appeared to find this challenging, *“erm… I think she’s probably just under halfway” (Sue).* Anna and Harry also put Ben “*right in the middle, cos we don’t know yet, it could go either way” (Anna)*, suggesting the inconclusive diagnosis put them in a liminal space:

*HARRY: “… and if it wasn’t for that potential you’d put him up there wouldn’t you [‘totally healthy child’], but yeah probably middle because there is that chance […] but we are taking into account the potential for things to go wrong, if it was just today and you didn’t know he had it you’d put him as completely healthy wouldn’t you.”*

Though Ben appears healthy, the underlying genotype stops Anna and Harry seeing him as so, with both concluding *“It’s the unknown.”* Multiple references to uncertainty, *“we don’t know yet”*, *“potential”, “could”, “chance”,* suggest the parents’ apprehension.

Liz and Guy were the only ones to thoroughly reject the diagnosis, *“[laughing] no question, I’d put her here [marks ‘completely healthy’]” (Guy).* Guy appeared to find it laughable that this was even being asked. However, by taking part in the interview, he was perturbed to realise that the picture was not as certain as he had thought, *“I mean […] do people mark here?* [‘serious condition’]*”*, *“I’m just trying to figure out [...] how could you say […]?”*.

Sadie marked Mia ‘completely healthy’, but also conceded *“you don’t know what’s round the corner for these kids, they’re doing fine now but in 5, 6 years they might not be”*. The uncertain future was also discussed by Molly and Jim. Molly quickly marked Ruby ‘completely healthy’, but Jim suggested it is less binary, *“[…] up towards this end”*. Despite her previous certainty, Molly acknowledged the *“chance”* of a less positive outcome:

*“[The doctor] said, ‘We still don’t know what 30 years, 40 years will look like, on her lungs’, so y’know it’s still keeping that in in the back of our mind all the time that there is that chance […] Especially cos they don’t know what the future could look like, it’s that uncertainty now, for this type of generation.”*

Molly notes that CFSPID’s long-term prognosis is unknown for both parents and professionals, which again may shake the traditional view of medicine as a certain institution.

#### 3.2.2. Tension of Idiosyncratic Forms of Certainty

Individuals created their own unique certainties to resolve the uncertainty of CFSPID. Dissonance arose between parents and children, professionals and parents, and within couples. For Sue, certainty appeared to be conferred by the prophylactic medical regimen that 3-year-old Tilly has been on since diagnosis:

*“It was gung-ho from the start and we’ve never really minimised anything we’ve never dropped her medication [...] all this prevention has stopped her getting ill, she could’ve been very poorly, we’ll never know will we.”*

Sue cannot be sure that intervention makes a significant difference, but by embracing diagnosis she is reassured by the medical world. Medication is seen to prevent illness, but also keeps Sue’s uncertainty and anxiety at bay. Sue sees the diagnosis as her battle (*“gung-ho”*), allowing her greater control over Tilly’s health. This is similar to Jim’s account.

In contrast, Guy, whose daughter Ava is not receiving treatment, rejects the diagnosis:

*“It doesn’t achieve anything, she’s not ill so you can’t say she’s got anything [laughs] […] There’s no effect on her life, no effect on our life—we were made aware of it, it was dealt with […] No so I don’t, er I dunno how you could say you’ve got, how people would say their child’s got CF or anything cos they haven’t.”*

Guy and Liz appear to attempt to understand the situation by vehemently rejecting diagnosis, appearing to find it unfathomable that anyone would see CFSPID as such. However, Molly’s story suggests this line cannot necessarily always be firmly drawn:

*[the daughter] used to say she couldn’t tidy up because she’s got cystic fibrosis […] she tried to use it as an excuse, but we’ve told her she’s got no excuse because look, a completely healthy child should do all the tidying up, shouldn’t they Ruby?”*

The power of labels is apparent in this example of a very young child embracing diagnosis. Molly and Jim reject the idea of Ruby seeing herself as ill, invoking ‘a healthy child’ to help her understand her identity. However, Ruby’s awareness of ‘having CF’ suggests that, at a young age, her sense of her situation potentially diverges from her parents’.

Different conceptualisations of CFSPID were not limited to individuals and families. Anna and Harry discussed the recent introduction of the CFSPID label, highlighting the dissonance between their views, and the views of healthcare professionals:

*ANNA: “Yeah they said ‘oh we’ve got a new term now for Ben now he’s a CFSPID’, I said it sounds like it’s an insult.”*

The account of the doctor casually mentioning this is at odds with the emotional weight of their interview. The introduction of the new label appears to have undermined the certainty that they had worked to achieve over the years. Anna’s creation of certainty involved using her understanding of a traditional medical model to understand and accept the diagnosis:

*“We do know that he’s got a faulty gene that does affect, it doesn’t work as well as ours does at getting the salt and the chloride around the body, we do know that he’s not completely healthy even though he looks it […] it’s a fact.”*

Anna appears more deterministic than Harry, who focuses more on the phenotype (*“surface level”*) than the genotype. Harry suggested that the impact of the diagnosis, and the discrepancy between their individual adaptations to CFSPID, has caused damage: *“I think it’s had lasting probably detrimental effects on the marriage […] the dynamic has changed”*.

Discrepancy was also experienced by Sadie and her ex-husband, with deleterious effects: *“He accused me of Munchausen by proxy” (Sadie).* Sadie’s ex-husband is able to reject CFSPID entirely in a way that would be impossible with a more obvious condition, leading to potential psychological harm. Again, because CFSPID is unseen and uncertain, Sadie was frustrated by inconsistency (*“moving the goalposts”*) from the professionals whom she looked to for guidance with shared custody. This exacerbated an already difficult situation:

*“The physio had to be done, and then it’s like ‘Well if dad doesn’t do the physio, as long as she’s having a little run round’, and I’m going ‘Which is it?!’”*

## 4. Discussion

This study furthers our understanding of the psychological impact of CFSPID designation following NBS for CF. This is a distinct situation in that parents’ expectations of health outcomes are being subverted by health care professionals. Although the NBS-positive children can often be asymptomatic, they have a definite condition. CFSPID moves this one step further by returning “information” which itself is unclear. Our results suggest that our participants’ experience of learning about their CFSPID, the uncertainty and, for some, ongoing health concerns had a significant negative psychological impact, in keeping with the only other qualitative study in this field [12]. For all parents, initial result communication caused heightened health risk and distress. Parents struggled to understand CFSPID, as it was incongruent with their preconceived illness and health care beliefs. There was tension between individuals’ distinct strategies to understand and process the CFSPID designation and manage uncertainty. Result communication should be changed to ensure that this does not happen.

We found that screening result communication was a key source of distress. This is a recognised problem in NBS [17] as delivering results to families is an unavoidable challenge for HCPs. The experiences of the parents in this study were similar to parents studied by Tluczek et al. [12,18] who experienced distress in the wait for further testing following positive NBS results. Though relatively shorter in our study, all parents still found this wait distressing. This echoes the work by Ulph et al. [6] who suggested that when parents’ information needs are not promptly met it can trigger anxiety and catastrophizing rumination. Appropriate management of this wait, bearing in mind the need to not offer false hope to families of infants with ‘proper’ CF, is key. Ensuring parents receive adequate, prompt, and accurate information, delivered with empathy, may minimise distress. HCPs should be supported with adequate resources to deliver this. This work fits with other studies in suggesting that telling parents not to go to the internet is unrealistic [6,12]. Indeed, it may fail to realise the psychological state triggered in parents on first awareness of a health risk and the need to take some form of “information gathering action” to lessen this undesirable state. Rather, it would appear that signposting parents to appropriate sites would be more realistic and beneficial for their psychological states.

Our findings fit with those of Tluczek et al. [12] in suggesting that anxiety triggered by the CFSPID result endures for parents. As such, they differ from parents who receive carrier results where anxiety dissipates after professional information [6]. This may be because there is extant information, whereas for CFSPID, parents could not be offered an adequately firm explanation of their child’s result, leaving them in limbo [8]. This may explain some parents’ continued belief that their child had CF, despite knowing about CFSPID and reporting satisfaction with the information and support received. A quantitative study may only have noted this latter point, whereas our study suggests the overall response is more complex.

La Pean and Farrell [19] suggested that initially misleading information following screening can leave a lasting impression, due to the primacy effect. In our study, parents’ perception of the initial message they received was that their child had CF. From our results it seems that the initial communication has a deep and profound impact on the parent’s subsequent beliefs with respect to their infant’s health risks. Schema theory [20], which posits that knowledge is arranged in groupings (schema) and that people try to fit new information into these schemas, may be relevant here. For some parents in our study, this led to anxiety over the potentially benign. This was reinforced by ongoing monitoring and, in some cases, prophylactic treatment (counter to European Cystic Fibrosis Society guidance [21]), echoing concerns that such approaches may over-medicalise and confer ‘the sick role’ [11,22]. Appropriate information at the outset and reassurance that their child is not at immediate risk and does not have CF is critical in moderating these parents’ responses to CFSPID. It is particularly important to stress that their child does not necessarily have classic CF, since parents will often be cared for in CF clinics and signposted to CF resources, as such it may not be an easy message for parents to assimilate. It is worth noting that UK guidance has recently changed such that a preparatory phone call to parents is recommended prior to NBS result delivery [21]. This may help to reduce the anxiety-provoking aspect of HCPs ‘turning up unannounced’, as described by the parents in this study. Though research which has captured pre-meeting phone call practice suggests there is still a high level of distress and that better preparing parents before NBS would be beneficial [6].

Results suggest that CFSPID subverts accepted ideas of health and illness, in giving parents a medical result which does not match their child’s observed phenotype. The results also suggest that parents may cope with this disparity by forming a strong belief in either the ‘healthy’ genotype or the ‘ill’ phenotype. Parents are generally unaware of the purpose or potential outcomes of NBS [17], less so the possibility of results of uncertain significance [2]. Appropriate explanation of this possibility, balancing pragmatism with reassurance that not all results are necessarily threatening, may help parents adapt to this new form of screening.

All parents were aware that CFSPID was a new classification. The designation was introduced in 2016 after a complex decision process by international stakeholders [8]. This process is not usually witnessed by laypeople, the names of diseases strongly influence our understanding of them and do not usually change. This may explain why most parents appeared to stick to the most salient aspect of the label, ‘CF’. In light of the possible effect of schemas [20], our results suggest that the names of inconclusive diagnoses must not misleadingly invoke diagnosis threat. Indeed, the importance of the label echoes findings by Tluczek [12] and the concerns of professionals in the original designation process [8], reflects the complexity of this issue.

We found that CFSPID causes uncertainty, corroborating extant concerns [8]. Results suggest that individuals, including HCPs as well as family, develop idiosyncratic mechanisms to attempt to form certainty. This is consistent with work by Dillon and Carson [23], who found that parents of children having further testing after a positive CF screen managed uncertainty in a range of ways. The range of mechanisms suggested by our results may be loosely mapped onto the options proposed by Massie & Gillam [11], parents ranged from managing their child as if they had CF to rejecting the diagnosis and thinking of them as a healthy carrier. Our results showed that parents in the same geographical location, with children with the same designation, vary widely in their treatment and perception of their children as ‘ill’ or ‘healthy’. Results suggest that many factors are involved here, including individual differences in health beliefs, parents’ relationships and support networks, and the children themselves, in addition to how the initial screening result and diagnostic assessment is experienced. All of these factors should be appreciated by the HCPs looking after the family.

Our results showed how tensions may arise between the views of parents and HCPs. Parents need support to manage the uncertainty of CFSPID, so that relationship damage is minimised. HCPs may be instrumental in managing uncertainty, through consistency and accuracy in the initial stages of diagnosis [23]. All of our parents reported positive experiences with HCPs, but there were some tensions between doctors’ and parents’ understanding of the CFSPID label, again emphasising the important role that illness labels play in adaptation. There is awareness that this is an uncertain situation not just for parents but for HCPs too [8], which is another aspect of CFSPID subverting the traditional medical model.

## 5. Strengths & Limitations

The interview schedule was designed with a clinician with considerable experience of working with parents with CFSPID and debates around management and a researcher with expertise in qualitative methods and research regarding NBS impact and communication. Drawing tasks, used to elicit responses conceptually, appeared to help parents reflect on how they viewed their child and their experience. In concordance with IPA methodology [16], the interviewer was trained to use the interview schedule and other materials flexibly and sensitively, and the conversations were driven by the participants. In fitting with IPA quality guidance [24], we have been transparent in order to enable readers to understand our process by providing a copy of the interview schedule, contextual information, and extensive process information. We have also focused on a defined in-depth topic and the data examples illustrate the depth and quality of data gained.

Meeting criteria set by Guba and Lincoln [25] specifically defined by Smith for IPA, we have illustrated rigour by giving the reader a sense of how themes are represented across the dataset as well as showing how experiences are idiosyncratic and differ, and yet also where they converge to illustrate a universal truth. We have allowed space and depth to explain each theme and subtheme and when presenting data, we have been careful to ensure that it is interpreted, illustrating the double hermeneutic, rather than positioned as a statement of fact.

The sample is in keeping with the guidance on IPA studies, where in the interest of ensuring depth-of-analysis, researchers are advised against large sample sizes. The analysis was supervised by an experienced qualitative researcher. Within the practical constraints of study recruitment and duration, the research has successfully gleaned insight into parents’ experiences with considerable variation in parental interpretation and contextual situations. The lack of fathers’ perspectives has been raised as a concern in newborn screening studies [1], yet a strength of this study is that a significant proportion were fathers. Furthermore, couples were interviewed together which enabled an appreciation of the considerable variance in the interpretation and impact of CFSPID and the tension this could cause in couples.

All research is affected by the bias of those who are willing to participate and give time to provide their accounts. Families were from the same geographical area of England and under the same health service, which may limit our understanding of CFSPID diagnoses in other areas. Families were majority white and relatively affluent and educated to a level that enabled them to engage in discussion of difficult issues, a feature similar to other studies concerning CF screening in particular [7,26]. Although this is commonly taken as a weakness in study evaluation, the fact that our participants struggled to comprehend and assimilate their experiences strengthens the need for communication guidance to ensure the wide range of parents who experience newborn screening are adequately informed and supported. Further qualitative research targeting more diverse groups would be beneficial in developing this.

## 6. Conclusions

The results from this detailed study illustrate the negative psychological impact of a CFSPID designation on parents. Factors leading to this may be unavoidable, but it is clear that with some straightforward changes in approach, the degree of impact could be lessened. A priority for change should be the first interaction with the family where the NBS result is presented accurately and empathetically, with a clear appreciation of what is to come. Subsequently, good communication is key and hopefully now that the CFSPID designation and management are established, CF HCPs will provide a more consistent message and advice to families.

## Figures and Tables

**Table 1:** Characteristics of parents.

*n* = 8 (families interviewed = 5)	
*Ethnicity*	
White British	8
*Gender*	
Female	5
Male	3
*Family unit*	
Lives with child’s father	7
Separated from child’s father	1
*Parents taking part in interview*	
Mother and father together	6
Mother only	2
*Highest educational attainment*	
Degree or higher	7
GCSE (school to 16 years)	1
*Number of Children*	
2	7
3	1
